# Spontaneous preterm birth prevention in multiple pregnancy

**DOI:** 10.1111/tog.12460

**Published:** 2018-01-28

**Authors:** Sarah R Murray, Sarah J Stock, Shona Cowan, Elizabeth Sarah Cooper, Jane E Norman

**Affiliations:** ^1^ MRC Centre for Reproductive Health Queen's Medical Research Institute University of Edinburgh Edinburgh EH16 4TJ UK; ^2^ Royal Infirmary Edinburgh Edinburgh EH16 4SA UK

**Keywords:** Arabin pessary, cerclage, multiple pregnancy, twins, vaginal progesterone

## Abstract

**Key content:**

- Twin pregnancies are associated with a three‐fold greater perinatal mortality than singleton pregnancies. Prematurity is a main contributor, with 50% of twin pregnancies delivering before 37 weeks and 10% delivering before 32 weeks of gestation.
- The aetiology of preterm delivery in twin pregnancies is likely multifactorial and different from that of singletons.
- Cervical cerclage reduces preterm birth rates in singletons but has mixed results in twins with some studies showing harm.
- The use of progesterone to prevent preterm birth in singletons has conflicting results and has not been proven to prevent preterm birth in twins. Studies continue to determine whether the cervical pessary is effective in preventing preterm birth in multiple pregnancies.
- There is a paucity of data available on the prevention of preterm birth in triplets/higher order multiples but similar principles to twin pregnancy apply.

**Learning objectives:**

- To review the burden of preterm birth in multiple pregnancy.
- To understand the methods available for preventing preterm birth in multiple pregnancies and the evidence surrounding the use of each one.
- To be aware of the use of the Arabin pessary.

## Introduction

Twin pregnancies are high‐risk and associated with increased perinatal morbidity and mortality.^1^ Multiple pregnancy is associated with adverse maternal outcomes including increased rates of pre‐eclampsia, pregnancy‐induced hypertension, maternal anaemia and venous thromboembolism.^1^ Compared to singleton newborns, newborn infants of twin pregnancies are also at increased risk of adverse outcomes including congenital anomalies, cerebral palsy, intrauterine growth restriction and stillbirth. Although only 3% of all live births are twin pregnancies,^1^ twin babies account for up to 15% of special care unit admissions.^2^ A 2009 survey conducted by the UK charity the Twin and Multiple Birth Association (TAMBA) found that in 44% of all twins born to the 1298 mothers interviewed, at least one baby entered special care.^3^

According to the 2014 MBRRACE‐UK (Mothers and Babies: Reducing Risk through Audits and Confidential Enquiries across the UK) report,^4^ the perinatal mortality rate in twins was three times higher than that of singleton pregnancies.^4^ Much of this perinatal mortality is driven by prematurity. In Scotland, 50% of twins are delivered preterm (at fewer than 37 weeks of gestation), with around 20% delivering before 34 weeks of gestation.^5^ Figures from the USA are similar: a 12‐fold higher preterm birth (PTB) rate of 56.6% was found in twins compared to 9.7% in singletons (odds ratio [OR] 12.8, 95% confidence interval [CI] 12.6–12.9).^6^

Despite attempts by the UK's National Institute for Health and Care Excellence (NICE) to reduce multiple pregnancy with the universal use of single embryo transfer (SET) during assisted reproduction technologies (ARTs),^7^ the UK twin pregnancy rate remains high among women undergoing ART, with up to 24% of successful in vitro fertilisation (IVF) procedures resulting in multiple pregnancy.^1^ As well as the increased risk of perinatal morbidity and mortality, the economic cost of healthcare provision for a twin baby is significantly higher than for singletons, and is quoted to be twice as high in the first 5 years.^8^

The James Lind Alliance (JLA) Preterm Birth Priority Setting Partnership (PSP) has prioritised the following question as the number one uncertainty in preterm birth: which interventions are most effective in predicting or preventing PTB? The JLA brings together clinicians, patients and carers to set research priorities in several aspects of obstetrics and gynaecology, including stillbirth and endometriosis.^9^

## Aetiology

The aetiology of spontaneous PTB in multiple pregnancies is likely to be multifactorial, different from singletons, and remains largely unknown. Different proposed pathophysiology mechanisms include intrauterine infection, cervical insufficiency and increased uterine stretch/distension. There is also increased secretion of mediators such as corticotrophin‐releasing hormone (CRH) from the larger placental mass, and factors produced by the maturing fetal lung such as surfactant protein‐A, which stimulates myometrial contractility and may contribute to preterm parturition.^10^

Twins are associated with a higher risk of obstetric intervention and therefore iatrogenic PTB is higher. Approximately one‐third of all premature deliveries in multiple pregnancies are medically indicated.^11^ This review focuses on the prevention of spontaneous PTB in twin pregnancies.

## Prediction of preterm labour in multiple pregnancy

Detailed descriptions of the methods of prediction of PTB in multiple pregnancy are out of the scope of this review, but the evidence surrounding the two most researched methods, cervical length and fetal fibronectin, is summarised below.

### Cervical length measurement

#### In asymptomatic women with a twin pregnancy

A 2010 meta‐analysis of 16 cohort/cross‐sectional studies (n = 3213)^12^ showed that in asymptomatic women with twins, a cervical length of <25 mm was associated with a 25% risk of delivery before 28 weeks of gestation. This review also found a cervical length of <20 mm at 20–24 weeks of gestation to be associated with a 42.4% risk of birth before 32 weeks, and a 62% risk of birth before 34 weeks. A subsequent systematic review^13^ upheld these findings and concluded that in asymptomatic women with a twin pregnancy, a cervical length measurement at 20–24 weeks of gestation was a good predictor of spontaneous PTB.

A recent systematic review of 1024 women with twins^14^ assessed repeated measures of a change in cervical length as a predictor of PTB, and found that the shortening of cervical length over time had a low predictive accuracy for preterm birth at fewer than 34 weeks of gestation.

The largest and most up‐to‐date individual patient data (IPD) meta‐analysis on the effect of gestational age and cervical length measurements in the prediction of PTB in twin pregnancies was published by Kindinger et al.^15^ in 2015 (n = 4409 twin pregnancies). The benefit of an IPD meta‐analysis is that it is a more robust method of combining and easily comparing studies, easily allowing subgroup analysis; for example, a short cervix group. This analysis consisted of 12 twin cohorts and found that when cervical length was <30 mm at 18 weeks of gestation, it was most predictive of birth at ≤28 weeks. Prediction of later spontaneous PTB (28–34 weeks) improved with cervical length measurements taken at later gestations (≥22 weeks). The authors concluded by recommending cervical length screening in twin pregnancies as a predictor of spontaneous PTB from 18 weeks of gestation.

In summary, in asymptomatic women with a twin pregnancy, current evidence supports the use of measuring cervical length from 18 weeks of gestation as a predictor of spontaneous PTB, but does not support repeated measures of cervical length.

#### In symptomatic women with a twin pregnancy

In a meta‐analysis of five combined cohort/cross‐sectional studies (n = 310), Conde‐Agudelo et al.^12^ showed that, in women with twin pregnancies and symptoms of spontaneous PTB, cervical length measurement had a low predictive accuracy for PTB at <34 weeks of gestation. However, the review concluded that the sample size was small and there is a paucity of evidence in the area of predicting PTB in twin pregnancies.

### Fetal fibronectin

#### In asymptomatic women with a twin pregnancy

NICE does not recommend the use of fetal fibronectin (fFN) in multiple pregnancies. A 2010 meta‐analysis^16^ summarising 11 studies of fFN use in asymptomatic twin pregnancy to predict PTB suggested only limited prediction accuracy, and better negative predictive rates than positive (6% risk of PTB before 34 weeks of gestation with a negative test compared to a 33% risk of PTB before 34 weeks with a positive test).

#### In symptomatic women with a twin pregnancy

The meta‐analysis by Conde‐Agudelo et al.^16^ synthesised the results of five studies using fFN to predict PTB in symptomatic women with a twin pregnancy. The authors concluded that fFN testing in multiple pregnancy was most accurate in women with symptoms of PTB (positive and negative likelihood ratios 85% and 75%, respectively, within 7 days of testing). However, although fFN testing is recommended for use in singleton pregnancies with symptoms of PTB if cervical length measurement is not available,^17^ it is not recommended in the NICE guideline for multiple pregnancy.

A combination of cervical length measurements and fFN may be a more accurate predictor of PTB. One small study^18^ (n = 155 twin pregnancies) reported that if fFN was positive and cervical length was <20 mm, then 54.4% of twins would deliver before 34 weeks of gestation, and this was significantly higher than the overall rate of PTB.

## Methods of preterm birth prevention

### Cervical cerclage

Cervical cerclage is a surgical technique to prevent PTB and has been described in the UK literature since 1902.^19^ Although cerclage is endorsed by NICE for use in singleton pregnancies, it is not recommended for use in multiple pregnancy as its effectiveness remains controversial.^11^ A subgroup of twins (n = 49 women) in a 2005 meta‐analysis^20^ investigating the use of cerclage showed an increase in prematurity and a trend towards harm. In multiple pregnancies, cerclage was associated with an increased risk of premature delivery (relative risk [RR] 2.15, 95% CI 1.15–4.01) and a trend towards an increased risk of perinatal mortality. However, this finding was not statistically significant, with wide confidence intervals likely reflecting the small sample size (RR 2.66, 95% CI 0.83–8.54).

A subsequent Cochrane review published in 2014^21^ examined five trials, of which two (n = 73 women) assessed history‐indicated cerclage and three (n = 55 women) assessed ultrasound‐indicated cerclage. This review found no benefit of cervical cerclage in reducing preterm delivery in twin pregnancies at fewer than 34 weeks of gestation (RR 1.16, 95% CI 0.44–3.36, four trials, n = 98 women). It also showed a trend towards harm with an increased risk of perinatal death, although this was not statistically significant (RR 1.74, 95% CI 0.92–3.28). There was no reduction in a composite of adverse neonatal outcome (RR 1.54, 95% CI 0.58–4.11). In the pre‐specified subgroup, ultrasound‐indicated cerclage was associated with an increased risk of low birthweight (RR 1.39, 95% CI 1.06–1.83, three trials, n = 98 women) and respiratory distress syndrome (RR 5.07, 95% CI 1.75–14.70, three trials, n = 98 women). The authors concluded that there was no evidence of the usefulness of cerclage in reducing the risk of PTB in twins, but that more research was needed because of a small number of trials, which each had a small number of patients. It is also important to note that no trials were identified that reported long‐term infant neurodevelopmental outcomes following cervical cerclage.

#### Ultrasound‐indicated cerclage in twin pregnancies

Following the Cochrane review, a further IPD meta‐analysis was published looking specifically at ultrasound‐indicated cerclage (cervical length ≤25 mm before 24 weeks of gestation) in twin pregnancies.^22^ Three randomised controlled trials (RCTs; n = 49 women) were identified. No significant differences in PTB rates before 34 weeks of gestation were found between cerclage and no cerclage in twin pregnancies with a trend towards harm (RR 2.19, 95% CI 0.72–6.63). Similar to the Cochrane review, the authors concluded that further large trials were necessary to determine the effectiveness of cerclage in twin pregnancies.

The latest retrospective cohort study was published in 2015 and involved 140 women with twin pregnancies.^23^ This showed that ultrasound‐indicated cerclage with a cervical length of ≤25 mm did not reduce the risk of preterm delivery before 34 weeks of gestation (adjusted OR [aOR] 0.37, 95% CI 0.16–1.1) nor was it associated with increased neonatal morbidity (aOR 0.7, 95% CI 0.4–1.2). In the pre‐specified subgroup analysis of women with a cervical length of ≤15 mm (n = 32 women), there was a significant reduction in the rate of PTB before 34 weeks of gestation (OR 0.42, 95% CI 0.24–0.81).

In summary, unlike singleton pregnancies, there appears to be no benefit of cervical cerclage in reducing PTB rates in multiple pregnancies. Although the study by Roman et al.^22^ reported a benefit in those with a short cervix (≤15 mm), the numbers in the study were small and further large trials are needed to address this question adequately.

A search on https://clinicaltrials.gov/ identified two currently recruiting studies comparing ultrasound and emergency cerclage in twin pregnancies with expectant management.^24^, ^25^ One trial^24^ is a multicentre, international RCT of physical exam‐indicated cerclage in twin gestations with a primary outcome measure of preterm delivery at fewer than 34 weeks of gestation. The other study^25^ is a single‐centre RCT of cervical cerclage versus expectant management for women with a twin pregnancy and a short cervix (≤25 mm), with a primary outcome of pregnancy prolongation. The ‘C‐STICH’ RCT^26^ of monofilament versus braided sutures for insufficient cervix is currently recruiting in multiple centres in the UK. Although this study only looks at singleton pregnancies, a similar trial in twin pregnancies would be appropriate after the results are published.

### Progesterone

#### Is it biologically plausible?

The use of progesterone in the prevention of PTB in both singleton and multiple pregnancies has been extensively investigated. Arguably, the use of progesterone is biologically plausible given that uterine quiescence is maintained throughout pregnancy by progesterone and progesterone receptor‐mediated inhibition of inflammation, which causes suppression of the contractile genes.^27^ Labour is thought to occur as a result of a functional withdrawal of progesterone.^28^ Anti‐progesterones such as mifepristone are used to stimulate abortion and induce labour. However, others have argued that progesterone levels are high during pregnancy, and progesterone receptors are fully occupied, hence the therapeutic benefit of adding further progesterone is unclear. Progesterone is available as an intramuscular injection of 17α‐hydroxylase caproate (only licensed in the USA), or vaginal progesterone (the only available progesterone product in the UK, but not licensed for prevention of PTB in the USA or Europe).

#### Use of progesterone in unselected twin pregnancies

A 2012 IPD meta‐analysis by Romero et al.,^27^ which investigated the use of vaginal progesterone in singletons, also included a subgroup of women with twins (n = 52 women). This revealed no reduction in PTB before 34 weeks of gestation when vaginal progesterone was used in twin pregnancies (RR 0.7, 95% CI 0.34–1.44). A subsequent IPD meta‐analysis by Schuit et al. (2015)^29^ upheld these main conclusions. The review included 13 trials (3768 pregnancies) of the use of progestogens in unselected twin pregnancies and the primary outcome was neonatal morbidity. Treatment with vaginal progesterone did not reduce the risk of adverse perinatal outcome (RR 0.97, 95% CI 0.77–1.2), and there were no significant differences for delivery before 32 weeks of gestation between the progesterone and the control groups (RR 0.91, 95% CI 0.68–1.2).^29^

A recent systematic review and meta‐analysis comparing progesterone, cerclage and the cervical pessary for prevention of PTB in unselected twin pregnancies found no reduction in PTB rates with any of the interventions.^30^ Some secondary outcomes were reduced with vaginal progesterone only (very low birth weight, [RR 0.71, 95% CI 0.52–0.98], and need for mechanical ventilation, [RR 0.61, 95% CI 0.45–0.82]).

One of the larger RCTs in the Schuit et al.^29^ IPD meta‐analysis, the ‘STOPPIT’ study (n = 500 women),^31^ performed a baby follow‐up study of the effect of prophylactic progesterone in twin pregnancy on childhood outcome.^32^ Performed through record linkage of childhood records, this study found no increased incidence of perinatal death (15 twins in the progesterone group versus 11 in the placebo group), congenital anomalies (OR 1.04, 95% CI 0.49–1.21) or hospitalisation (OR 0.97, 95% CI 0.71–1.33) of those exposed to progesterone versus placebo. The authors concluded there was no evidence of a detrimental or beneficial impact on health and developmental outcomes at 3 and 6 years of exposure to progesterone in utero.

#### Use of progesterone in twin pregnancies in women with a short cervix

Debate still exists as to whether progesterone is effective in singleton pregnancies of women with a short cervix. The Schuit et al.^29^ IPD meta‐analysis published in 2015 included a subgroup analysis of women with a twin pregnancy and a short cervix of ≤25 mm (n = 116 women). Among this subgroup, vaginal progesterone provided a protective effect over the control with regards to adverse perinatal outcome (RR 0.57, 95% CI 0.47–0.70). However, the number of women in the subgroup was small, thus limited conclusions can be inferred. The primary outcome in this study was neonatal morbidity, therefore differences in PTB in the subgroup of women with a short cervix ≤25 mm were not investigated in this review.

In summary, there is no benefit of universal vaginal progesterone to reduce PTB rates in multiple pregnancies. One meta‐analysis showed a benefit in adverse perinatal outcome in a subgroup of women with a short cervix ≤25 mm, suggesting it may be useful in this group, but the numbers in the study were small and further research is needed. There appears to be no long‐term harm caused to infants exposed to progesterone in utero.^32^ The NICE guidelines for multiple pregnancy currently followed by UK practitioners do not promote the routine use of cervical cerclage or progesterone for the prevention of PTB in multiple pregnancies (see Box 1).

Box 1 **Preterm birth prevention in multiple pregnancy from the National Institute for Health and Care Excellence guideline 2011^1^**

Do not use the following interventions (alone or in combination) routinely to prevent spontaneous preterm birth in twin or triplet pregnancies:

- Bed rest at home or in hospital
- Intramuscular or vaginal progesterone
- Cervical cerclage
- Oral tocolytics

### Cervical pessary

**Figure 1:**
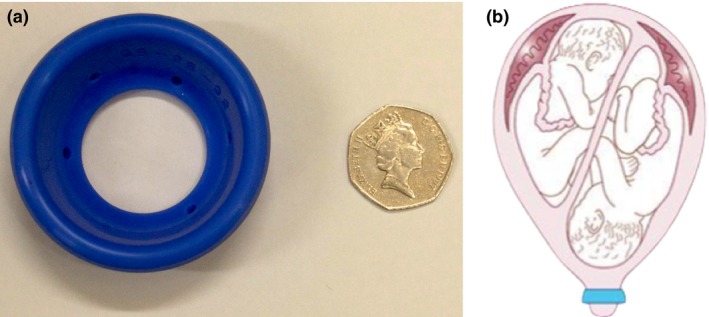
a) The Arabin cervical pessary; b) the position of the Arabin cervical pessary.

The Arabin pessary is a cervical pessary used to prevent PTB. It is a flexible silicon ring with a smaller inner diameter that encompasses the cervix, aiming to tilt it posteriorly and provide cervical support.^33^ It is usually inserted at around 18–22 weeks of gestation, and in twin pregnancies is removed before 36 weeks. The Arabin cervical pessary and its correct positioning are shown in Figure 1. Available evidence surrounding the use of the cervical pessary in twin pregnancies is conflicting and is discussed here in terms of unselected twin pregnancies and twins in women with a short cervix.

#### Use of the cervical pessary in unselected twin pregnancies

Two RCTs looking at the use of the cervical pessary in unselected twin pregnancies have been performed. The largest, by Nicolaides et al.^34^, which involved 1180 women who received a cervical pessary versus expectant management, found no difference in the rate of PTB before 34 weeks of gestation (RR 1.05, 95% CI 0.79–1.41).

The ProTWIN study^35^ was an RCT of 808 unselected twin pregnancies. This study found no difference in PTB before 32 weeks of gestation (10% versus 12%, RR 0.86, 95% CI 0.65–1.15) and no reduction in adverse perinatal outcome (RR 0.98, 95% CI 0.69–1.39).

#### Use of the cervical pessary in twin pregnancies with a short cervix

The PECEP‐Twins trial,^36^ published in 2016, is the only RCT to focus on twins in women with a short cervix (defined as ≤25 mm). In this study, 137 women with a short cervix were randomly assigned to cervical pessary or expectant management groups. The rate of PTB before 34 weeks of gestation was reduced in the pessary group compared with the expectant management group (RR 0.41, 95% CI 0.22–0.76). In a post hoc subgroup analysis of the study by Nicolaides et al.,^34^ the incidence of PTB before 34 weeks of gestation among 106 women with a cervical length of <25 mm was not significantly different between the pessary group and the expectant management group (31% versus 26%, RR 1.2, 95% CI 0.8–1.8). In contrast, subgroup analysis of the ProTWIN study,^35^ which used a cervical length cut‐off of <38 mm, reported a reduction in PTB before 32 weeks of gestation (16.2% versus 39.4%; RR 0.41, 95% CI 0.22–0.76).

**Figure 2:**
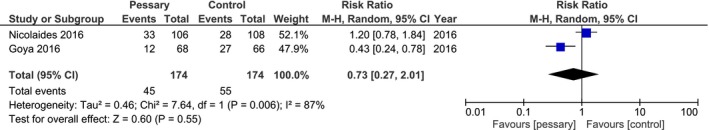
Risk of preterm birth <34 weeks of gestation in twin pregnancies with a cervical length <25 mm.^37^

In a systematic review and meta‐analysis, Saccone et al.^37^ combined the results of the three trials of cervical pessary use for the prevention of PTB of twins of women with a short cervical length, and a forest plot was produced (Figure 2). It is important to note here that because the Nicolaides et al. and ProTWIN studies used different cervical length cut‐offs (<25 mm and <38 mm, respectively), they were not directly comparable in the meta‐analysis and therefore were not powered to detect a difference in PTB in these subgroups. The review concluded that use of the Arabin pessary in twin pregnancies of women with a short cervix may not prevent PTB or improve perinatal outcome.

In summary, evidence for the use of the cervical pessary for the prevention of PTB in twins is conflicting, though there is some evidence to suggest it may be useful in twin pregnancies of women with a short cervix. The STOPPIT 2 RCT^38^ is currently recruiting in the UK to help address this paucity of evidence. This RCT aims to resolve the uncertainty surrounding whether or not the Arabin pessary reduces spontaneous PTB in twins of women with a short cervix (<30^th^ centile, which equates to 35 mm^15^). The cervical pessary is not currently routinely used in clinical practice outside of the research setting.

### Other methods of preterm birth prevention

As stated in Box 1, the following methods of PTB prevention in multiple pregnancies are not endorsed by NICE. The evidence is summarised below:

#### Bed rest and uterine monitoring

In the past, bed rest was used as a method of PTB prevention for both singleton and twin pregnancies. A 2010 Cochrane review^39^ comparing hospitalisation and bed rest against expectant management summarised the results of six trials (n = 600 women). It found no benefit of this intervention, but did find an increased risk of PTB before 34 weeks of gestation (OR 1.84, 95% CI 1.01–3.34). Similarly, a 1995 meta‐analysis^40^ of six trials on the use of home uterine monitoring showed no reduction in PTB. Thus, neither of these interventions have been endorsed by NICE.

#### Prophylactic tocolytics

For singleton pregnancies, the RCOG's Green‐top Guideline^41^ (GTG) supports the use of tocolysis for the completion of corticosteroids or an in utero transfer, but not for PTB prevention. Similarly, for multiple pregnancy, the GTG states that there is insufficient evidence for its use in the prevention of preterm labour. A 2005 Cochrane review^42^ of five trials (n = 344 women) found no reduction in PTB before 34 weeks of gestation with the use of tocolytics (RR 0.47, 95% CI 0.15–1.50).

In summary, the use of prophylactic tocolytics to prevent preterm labour in multiple pregnancy is not recommended.

## Conclusion

There is a lack of effective, evidence‐based interventions for the prevention of PTB in twin pregnancies. There is limited evidence for the use of vaginal progesterone and cervical cerclage, and the cervical pessary is currently only used within a research setting. There are no reported trials comparing the effectiveness of each of these interventions against each other, whether in isolation or in combination.^43^ Likewise, although fFN and cervical length scanning may be beneficial in predicting PTB in twins either alone or in combination, no reliable evidence supports the use of any one predictor. Research is needed to further evaluate the benefit of the cervical pessary and the use of cervical cerclage in twins of women with a short cervix. A recent article by Stock et al.^43^ concludes by advising clinicians to share with women the uncertainty of methods to prevent PTB in multiple pregnancy, and offer the opportunity to participate in clinical trials.

### Disclosure of interests

JEN has research grants from government and charitable organisations (Tommy's, the baby charity, the Medical Research Council and National Institute of Health Research [NIHR]) for preterm birth prevention (including in twins). The University of Edinburgh receives also funding for her contribution to a data monitoring committee for a preterm birth study run by GlaxoSmithKline. SRM, SJS, SC and ESC have no conflicts of interest.

### Contributions to authorship

SRM and JEN instigated the article. SRM researched and wrote the article. SJS, ESC, JEN and SC edited the article. All authors approved the final version.
